# RETRACTION: Sternal Wound Infections Following Internal Mammary Artery Grafts for a Coronary Bypass: A Meta‐Analysis

**DOI:** 10.1111/iwj.70512

**Published:** 2025-04-18

## Abstract

**RETRACTION:** LiM. , YuZ. , ChenQ. , ZhaoQ. , ChenX. , LeiC. , WangX. , and YangR. , “Sternal Wound Infections Following Internal Mammary Artery Grafts for a Coronary Bypass: A Meta‐Analysis,” International Wound Journal 21, no. 1 (2024): e14349, 10.1111/iwj.14349.37596778 PMC10781594

The above article, published online on 18 August 2023, in Wiley Online Library (http://onlinelibrary.wiley.com/), has been retracted by agreement between the journal Editor in Chief, Professor Keith Harding; and John Wiley & Sons Ltd. Following an investigation by the publisher, all parties have concluded that this article was accepted solely on the basis of a compromised peer review process. The editors have therefore decided to retract the article. The authors did not respond to our notice regarding the retraction.

**RETRACTION:** M. Li , Z. Yu , Q. Chen , Q. Zhao , X. Chen , C. Lei , X. Wang , and R. Yang , “Sternal Wound Infections Following Internal Mammary Artery Grafts for a Coronary Bypass: A Meta‐Analysis,” International Wound Journal 21, no. 1 (2024): e14349, 10.1111/iwj.14349.37596778 PMC10781594

The above article, published online on 18 August 2023, in Wiley Online Library (http://onlinelibrary.wiley.com/), has been retracted by agreement between the journal Editor in Chief, Professor Keith Harding; and John Wiley & Sons Ltd. Following an investigation by the publisher, all parties have concluded that this article was accepted solely on the basis of a compromised peer review process. The editors have therefore decided to retract the article. The authors did not respond to our notice regarding the retraction.

